# Profiling of infection specific mRNA transcripts of the European seabass *Dicentrarchus labrax*

**DOI:** 10.1186/1471-2164-10-157

**Published:** 2009-04-10

**Authors:** Elena Sarropoulou, Pilar Sepulcre, Laura Poisa-Beiro, Victoriano Mulero, José Meseguer, Antonio Figueras, Beatriz Novoa, Vasso Terzoglou, Richard Reinhardt, Antonios Magoulas, Georgios Kotoulas

**Affiliations:** 1 Institute of Marine Biology and Genetics, Hellenic Center of Marine Research, PO Box 2214, 710 03 Iraklio, Crete, Greece; 2 Department of Cell Biology and Histology, Faculty of Biology, University of Murcia, 30100 Murcia, Spain; 3 Instituto de Investigaciones Marinas, Consejo Superior de Investigaciones Científicas (CSIC), Eduardo Cabello, 6 36208, Vigo, Spain; 4 Max-Planck Institute for Molecular Genetics, Ihnestrasse 63–73, 14195 Berlin-Dahlem, Germany

## Abstract

**Background:**

The European seabass (*Dicentrarchus labrax*), one of the most extensively cultured species in European aquaculture productions, is, along with the gilthead sea bream (*Sparus aurata*), a prospective model species for the Perciformes which includes several other commercially important species. Massive mortalities may be caused by bacterial or viral infections in intensive aquaculture production. Revealing transcripts involved in immune response and studying their relative expression enhances the understanding of the immune response mechanism and consequently also the creation of vaccines. The analysis of expressed sequence tags (EST) is an efficient and easy approach for gene discovery, comparative genomics and for examining gene expression in specific tissues in a qualitative and quantitative way.

**Results:**

Here we describe the construction, analysis and comparison of a total of ten cDNA libraries, six from different tissues infected with *V. anguillarum *(liver, spleen, head kidney, gill, peritoneal exudates and intestine) and four cDNA libraries from different tissues infected with Nodavirus (liver, spleen, head kidney and brain). In total 9605 sequences representing 3075 (32%) unique sequences (set of sequences obtained after clustering) were obtained and analysed. Among the sequences several immune-related proteins were identified for the first time in the order of Perciformes as well as in Teleostei.

**Conclusion:**

The present study provides new information to the Gene Index of seabass. It gives a unigene set that will make a significant contribution to functional genomic studies and to studies of differential gene expression in relation to the immune system. In addition some of the potentially interesting genes identified by *in silico *analysis and confirmed by real-time PCR are putative biomarkers for bacterial and viral infections in fish.

## Background

The European seabass *Dicentrarchus labrax *is one of the most extensively aquacultured fish species in the Mediterranean, resulting in steadily increasing pressure on producers. Consequently, it is important to acquire new techniques and knowledge in order to improve aquaculture practices. Detailed information concerning growth, health, disease resistance and flesh quality benefit from the molecular as well as from the physiological point of view can provide illuminating new findings leading to improved aquaculture techniques. Several efforts have been made up till now to enrich the genomic resources in aquaculture production in the Mediterranean (chiefly for the gilthead sea bream *Sparus aurata *and for the European seabass *Dicentrarchus labrax*), e.g. Marine Genomics Europe (Network of Excellence) (CT-2003-505403), [1-5] as well as in the Atlantic (e.g. Atlantic halibut *Hippoglossus hippoglossus*, Salmon *Salmo salar*) e.g. [6-13]. These studies focused mainly on non-challenged tissues in order to obtain a first unigene catalogue. Aquaculture production however is affected by viral and pathogenic bacteria, particularly in respect of *D. labrax *which has been shown to be the species most sensitive to pathogenic bacteria such as *Vibrio anguillarum *[14] and to viral infections such as Nodavirus [15,16]. There are several commercial vaccines which provide protection against infection from *V. anguillarum *although the mechanism of immune response still remains unknown. Nodavirus can cause massive mortalities [17] and cannot be controlled so far because the production of commercial vaccines here is still in its infancy. In the present study we have generated a collection of EST sequences from tissues of European seabass infected with *V. anguillarum *and Nodavirus. Within this collection we were able to isolate immune relevant genes, and have gone on to compare gene expression in different tissues after viral and pathogenic bacteria infection. Additionally we determined *in silico *differential expression between the two infections. In this context the construction and analysis of a total of ten cDNA libraries are described; six cDNA libraries were from tissues of the European seabass infected with *V. anguillarum *(liver, spleen, head kidney, peritoneal exudate, gill and intestine) with peritoneal exudate, gill and intestine as target organs for *V. anguillarum *infections, and four cDNA libraries were from tissues of the European seabass infected with Nodavirus (liver, spleen, head kidney and brain) with the brain as target organ of the virus. Comparisons between the predicted European seabass peptide data set and the zebrafish, medaka, stickleback, tetraodon and human proteomes were performed. Genes showing *in silico *differential expression between Nodavirus infection and *V. anguillarum *infection were further analysed by real-time PCR.

## Results

### Summary of ESTs from the cDNA libraries infected with Nodavirus and V. anguillarum

**Figure 1:**
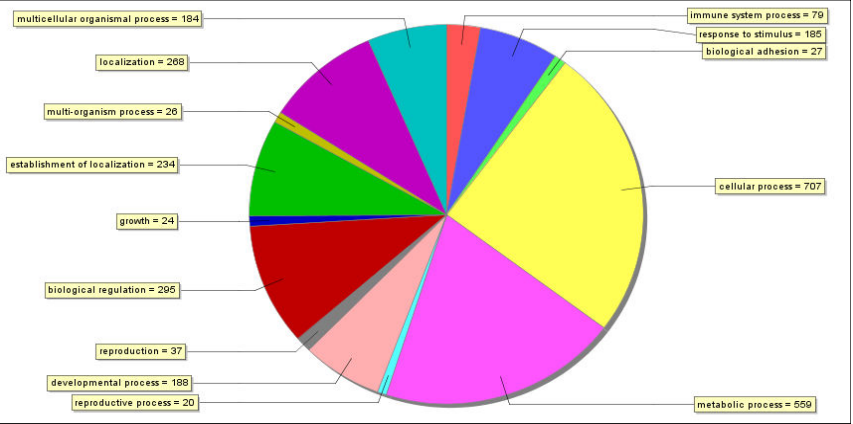
**Summary of GO category Biological process of unique ESTs obtained from seabass cDNA libraries infected with Nodavirus and *Vibrio anguillarum***.

The amplified libraries contained insert size from approximately 0.5 to 2.0 kb. Single pass sequencing was performed resulting in 9605 high quality sequences. All sequences were submitted to the EST database (dbEST  with the accession numbers **FK939975 – FK944381**, **FL484477 – FL488763 **and **FL501471 – FL502381**. A set of 3075 unique sequences was generated. Among the unique sequences (3075) [see Additional file 1] 371 [12%, see Additional file 2] sequences contained Simple Sequence Repeats (SSR). Cluster analyses performed for each library separately (Table 1a and Table 1b) revealed redundancy rates which varied from 72% (28% unique sequences) in intestine cDNA library infected with *V. anguillarum *to 37% (63% unique sequences) in spleen cDNA library infected with *V. anguillarum *(Table 1a). The set of unique EST sequences was annotated with Blast2GO which carries out BLASTX searches and attempts to assign function and GO classification. Out of the 3075 unique EST sequences submitted to GO2Blast for annotation and GO classification, 1521 sequences fell into 14 categories of biological process function at GO annotation level 2 (Fig. 1), where two categories, cellular process and metabolic process, were predominant. The category "immune system process" was represented by 79 transcripts.

**Table 1:** Summary of sequences derived of cDNA libraries of *D. labrax *tissue infected with V. anguillarum (a) and Nodavirus (b)

**A**					
**Tissue**	**singletons**	**contigs**	**unique**	**total sequences**	**% of unique sequences**

Liver	503	190	693	2140	32.38
Spleen	326	38	364	651	63.14
Kidney	266	66	332	911	36.24
Peritoneal exudate	386	103	489	827	59.13
Gill	92	15	107	343	31.19
Intestine	88	4	92	326	28.22

**B**					

**Tissue**	**singletons**	**contigs**	**unique**	**total sequences**	**% of unique sequences**

Liver	253	127	380	1126	33.75
Spleen	698	118	816	1284	63.55
Brain	617	41	658	1099	59.87
Kidney	321	55	376	934	40.26

### EST matches with known function

Out of the 3075 EST sequences, 1246 (~ 41%) had a positive hit after submission to BLASTX database search. Among those EST sequences with a known function, 128 homologues were found to be involved in the immune response and 79 of these were grouped into the GO category "immune system process". The remaining 49 transcripts were manually determined to be involved in the immune response (see Additional file 3). Immune related transcripts isolated for the first time for seabass amounted to 115 (Table 2). Among transcripts of interest, the transcript encoding for an important antimicrobial protein, hepicidin, was isolated. Aligning EST sequences grouped into one contig can provide additional data. In the case of hepcidin it is probable that different isoforms are grouped together. Alignments of other cDNA sequences either showed alternative polyadenylation or they showed *in silico *polymorphism of microsatellite DNA as for instance the transcript coding for cysteine-rich protein 1-I (see Additional file 4).

**Table 2:** Transcripts isolated for the first time in *D. labrax *and grouped to the GO category" immune system process"

**Contig ID**	**BLASTX Hit**	**Accession number**
Contig_265, Contig_3	alpha globin	Q9PVM4 BAA86218
Contig_681	alpha-1-microglobulin bikunin precursor	CAA45294
Contig_3061	b-cell leukemia lymphoma 6	XP_001340785
Contig_1838	bcl2 adenovirus e1b 19 kda interacting protein 3	NP_001012245, AAR83676
Contig_276	beta-2 microglobulin	ABB60035, ABB60037
Contig_773	beta-2 microglobulin precursor	AAC64994
Contig_2731	blood thirsty	AAX12162
Contig_2210	blood thirsty	XP_699830
Contig_1617	c1 inhibitor	NP_001117851, CAD58653
Contig_936	cathepsin s	AAQ01147
Contig_2779	cc chemokine	AAY79324
Contig_616	ccaat enhancer binding protein (c ebp)beta	BAB40971
Contig_1810	cd59-like protein 2	NP_001117969, AAT94063
Contig_1441	cell division cycle 42	NP_956159, AAH48035, AAH75761, AAX20139, CAM56524, AAI64988
Contig_2676	chemokine (c-c motif) ligand 13	BAC20610
Contig_2058	chemokine (c-c motif) ligand 21b	AAT52146, ABA54959
Contig_241	chemokine (c-c motif) ligand 25	ABC69050
Contig_2858	chemokine (c-x-c motif) ligand 12b (stromal cell-derived factor 1)	NP_840092, AAN64414, AAS92649, AAI09418
Contig_524	chemokine (c-x-c motif) ligand 9	ABC69049
Contig_627	chemokine (c-x-c motif) receptor 4	ABP48751
Contig_525, Contig_1672	chemokine cxc-like protein	ABC69049
Contig_983	complement c4-2	CAD45003
Contig_2306	complement component 1 q subcomponent	ABV57766
Contig_513	complement component 1 qb chain	XP_001110783
Contig_986	complement component 5	BAC23058
Contig_1044	complement component 7	BAA88899
Contig_2558	complement component alpha polypeptide	NP_001118096, CAH6548
Contig_1413	complement component c3	BAA88901
Contig_1114	complement component c4	CAD45003
Contig_2843	complement component c5-1	BAC23057
Contig_2534, Contig_2600	complement component c9	P79755, AAC60288
Contig_1499	complement component factor h	NP_001117882, CAF25505
Contig_1496	complement component gamma polypeptide	NP_001117880, CAF22027
Contig_927	complement component1, q gamma polypeptide	XP_544508
Contig_2432	complement component beta subunit	Q9PVW7, BAA86877
Contig_2605	complement component q subcomponent binding protein	EDM05067
Contig_1536	complement component r subcomponent	AAR20889
Contig_868	complement factor b	CAD21938
Contig_1607	complement factor d preproprotein	XP_001117186
Contig_2013	complement factor h	NP_001117876, CAF05664, CAF05665
Contig_1481	complement factor h-related 1	AAA92556
Contig_1375	cornichon homolog	O35372, AAC15828
Contig_1198	c-reactive protein	NP_999009, O19062 BAA21473
Contig_1657	c-type lectin	BAE45333
Contig_1596	cu zn superoxide dismutase	AAW29025
Contig_395	deah (asp-glu-ala-his) box polypeptide 16	NP_956318, AAH45393, AAI65206
Contig_2814	ets-1 transcript variant ets-1 delta(iii-vi)	AAY19514
Contig_626	ferritin heavy chain	NP_001117129, P49946, AAB34575
Contig_280	fth1 protein	CAL92185
Contig_2392	g-protein couplededg6	NP_001112363
Contig_2662	heat shock 10 kda protein 1 (chaperonin 10)	AAV37068
Contig_2975	heat shock 70 kda protein 4	AAH65970
Contig_2939	heme oxygenase1	ABL74501
Contig_275	hemoglobin alpha chain	CAP69820
Contig_731	Hephaestin	NP_579838
Contig_1713	hypoxanthine phosphoribosyltransferase 1	NP_001002056, AAH71336
Contig_1745, Contig_1876	integrin beta 2	BAB39130, NP_990582, CAA50671
Contig_2452	interleukin 18 receptor accessory protein	XP_001371334
Contig_2261	interleukin 1 type i	XP_416914
Contig_1718	interleukin 1 type ii	NP_001015713, AAH89644
Contig_1506	interleukin 2 receptor gamma chain	CAJ38407
Contig_1835	interleukin enhancer binding factor 3	AAH47175
Contig_966	interleukin-1 receptor type ii	ABP99035
Contig_852	interleukin-1 receptor type ii	CAL30143
Contig_2196	loc559360 protein	AAI51869
Contig_2044	macrophage migration inhibitory factor	ABG54276
Contig_1348	major histocompatibility complex class i a chain	BAD13369
Contig_17	mflj00348 protein	BAD90390
Contig_889	mhc class i alpha antigen	ABB04088
Contig_1349	mhc class i antigen	BAD13366
Contig_2797	mitochondrial ribosomal protein s18b	NP_001106610, AAI52129, AAI55448
Contig_3008	natural resistance-associated macrophage protein	AAG31225
Contig_576	neurofibromatosis 1	AAD15839
Contig_1716	novel protein vertebrate complement component 3	NP_001116778, CAQ13357
Contig_1327	otuubiquitin aldehyde binding 1	NP_001002500, AAH76301
Contig_2661	Proteasome activator subunit 1 (pa28 alpha)	ABE60902, ABK41199
Contig_1948	protein kinase alpha	AAI51472
Contig_2319	protein tyrosinereceptorc	XP_547374
Contig_2009	purinergic receptorg-protein13	XP_001516794
Contig_1715	rhamnose binding lectin	NP_001117668, BAA92256
Contig_934	ribosomal protein s19	P61155, AAP20214
Contig_2680	sam domain- and hd domain-containing protein 1	XP_001097562
Contig_684	serum amyloid p-component	P12246 AAA40093, CAA34774, AAH61125, AAY88178, BAE25796, BAE38344, EDL39002
Contig_2165	sffv proviral integration 1	NP_035485
Contig_1473	sh2 containing inositol-5-phosphatase	XP_687502
Contig_2675	skin mucus lectin	BAD90686
Contig_840	strawberry notch homolog 2	EDL31603
Contig_1540	tnf superfamily member 14	NP_001118039, ABC84585
Contig_469	transcription factor 3 isoform cra_b	NP_571169, CAA54305
Contig_2299	transforming growth beta receptor ii (70 80 kda)	XP_534237
Contig_2692	trypsin 10	BAF76146
Contig_1814	vascular endothelial growth factor	NP_001038320, AAY89335
Contig_1660	x-box binding protein 1	AAQ08005

### Similarity relationships

**Figure 2:**
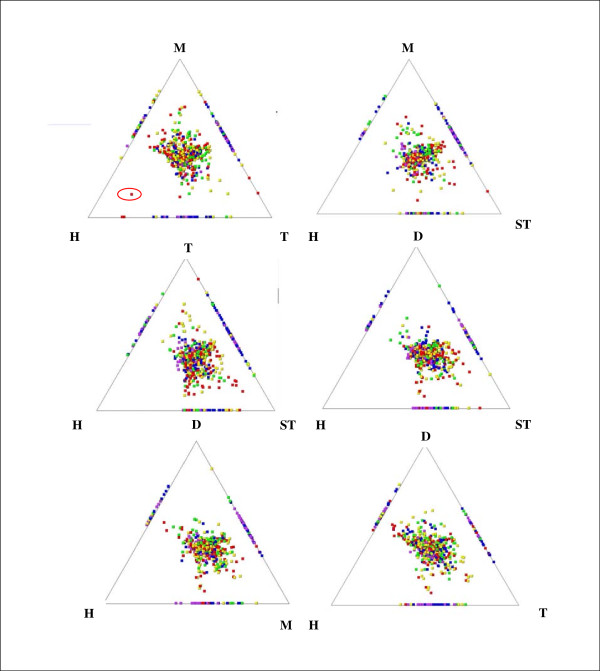
**Similarity of *D. labrax *ESTs to the proteomes of *Homo sapiens *(H), *Danio rerio *(D), *Oryzias latipes *(M), *Gasterosteus aculateus *(ST) and *Tetraodon nigroviridis *(T)**. SimiTri plots show the graphical similarity i) between putative *D. labrax *peptides and *H. sapiens*, *O. latipes *and *T. nigroviridis*, cytochrome b is circled in red ii) between putative *D. labrax *peptides and *H. sapiens*, *O. latipes *and *G. aculateus*, iii) between putative *D. labrax *peptides and *H. sapiens*, *D. rerio *and *T. nigroviridis*, iv) between putative *D. labrax *peptides and *H. sapiens*, *D. rerio, G. aculateus*, v) between putative *D. labrax *peptides and *H. sapiens*, *D. rerio, O. latipes*, as well as vi) between putative *D. labrax *peptides and *H. sapiens*, *D. rerio, T. nigroviridis*.

**Figure 3:**
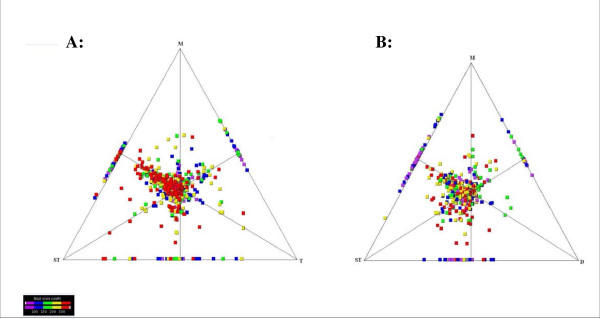
**Similarity of *D. labrax *ESTs to the proteomes of *Danio rerio *(D), *Oryzias latipes *(M), *Gasterosteus aculateus *(ST) and *Tetraodon nigroviridis *(T)**. SimiTri plots show the graphical similarity **A**: between putative *D. labrax *peptides and *G. aculateus, O. latipes *and *T. nigroviridis*, **B**: between putative *D. labrax *peptides and *G. aculateus*, *O. latipes *and *D. rerio*.

Figs. 2 and 3 show SimiTri representation of predicted seabass transcripts compared to *Danio rerio, Homo sapiens, Oncorhynchus mykiss, Gasterosteus aculateus *and *Tetraodon nigroviridis *proteomes. Of 3075 isolated unique transcripts 1040, 1051, 1122 1159, 1103 had Blast hits with a score > 50 against the *H. sapiens, D. rerio, O. mykiss, G. aculateus and T. nigroviridis *protein databases respectively.

### Expression analysis

**Figure 4:**
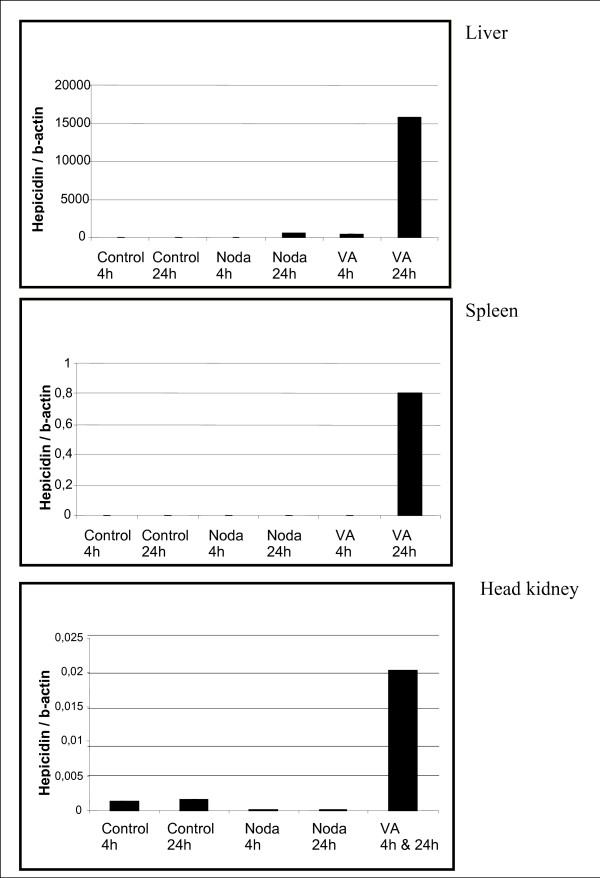
**Hepicidin precursor expression index of liver, spleen, and head kidney infected with Nodavirus or with *V. anguillarum *for 4 h and 24 h**. Infection for 4 h and 24 h of head kidney with *V. anguillarum *is pooled as not enough material was available. Each bar represents the mean of two technical duplicates of cDNA originating out of three individuals pooled prior to RNA extraction.

**Figure 5:**
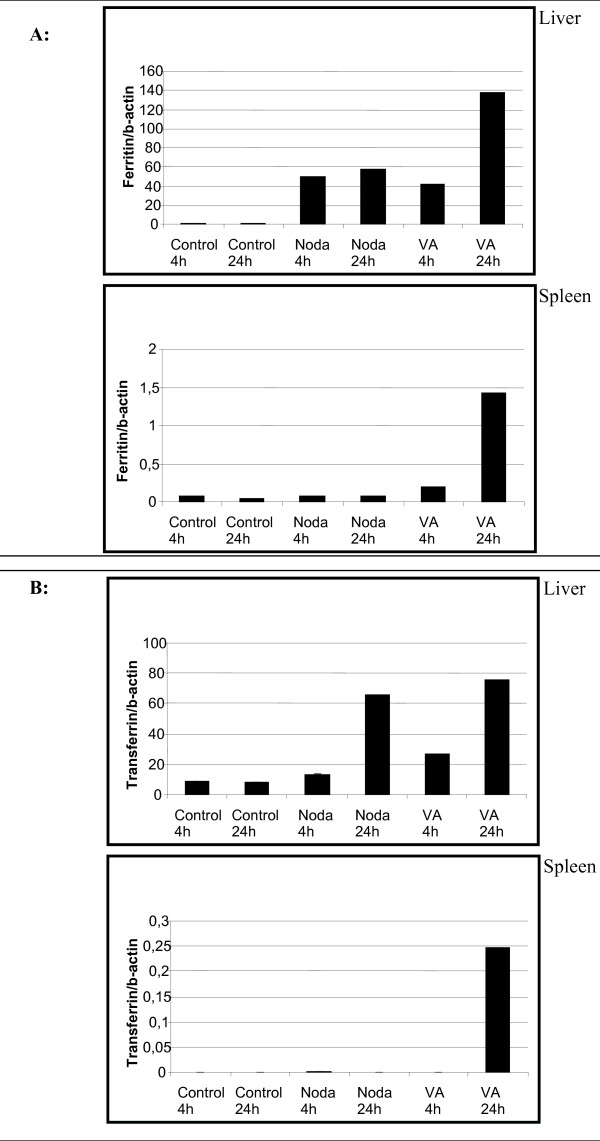
**(A) Ferritin expression index of liver and spleen infected with Nodavirus or with *V. anguillarum *for 4 h and 24 h**. **(B) **Transferrin expression index of liver and spleen infected with Nodavirus or with *V. anguillarum *for 4 h and 24 h. Each bar represents the mean of two technical duplicates of cDNA originating out of three individuals pooled prior to RNA extraction.

The test to compare multiple cDNA libraries with each other [22] revealed that the genes with the value > 6 of the test statistic R can be confidently considered as genes with true variation with the slope of 1.081 and are therefore not significantly different from -1 at the 5% level (see Additional file 6). The hits with R > 6 are in total 109 out of 2234 contigs resulting from EST sequences of liver, spleen and head kidney infected with Nodavirus and *V. anguillarum*. The list of the 109 transcripts with R > 6 and their putative homologues are shown in Additional file 5. It is interesting to note that although most transcripts were abundantly expressed in both bacterial and viral infected tissues, not all of them could be considered as specific markers of a specific infection. For example, fructose-1,6-biphosphate aldolase A, hepcidin, apolipoprotein A1 precursor, ferritin heavy chain and chemokine receptor 4 transcripts were found in *V. anguillarum*-infected tissues, though rarely in Nodavirus-infected tissues (see Additional file 5). Conversely, fructose-1,6-biphosphate aldolase B and 14 kDa apolipoprotein transcripts were frequently observed in Nodavirus infected tissues compared with *V. anguillarum*-infected tissues. The above results were further validated by determining the expression of putative markers for each infection in key tissues using real-time PCR. Here also control tissues were included in order to determine the expression of untreated fish. The real-time PCR confirmed the results obtained with the *in silico *analysis for selected genes. Taking into account the fold inductions of the real-time PCR experiments the correlations between *in silico *and qPCR are uniform. For instance the transcript for hepicidin precursor revealed *in silico *(R = 298.16) high expression only in liver tissues infected with *V. anguillarum*. The real-time PCR results show higher expression in all three tissues infected with *V. anguillarum*. However fold induction in liver is 20,000 times more than in spleen tissue, therefore theoretically 20,000 more cDNA clones had to be sequenced to obtain the sequence for hepicidin precursor in spleen infected with *V. anguillarum*. This correlation of high fold induction with *in silico *results can be observed for each transcript examined in this study. Thus, while the mRNA levels of hepcidin were found to increase considerably 24 h post-infection in the liver, spleen and head kidney of *V. anguillarum*-infected fish, they increased only slightly in Nodavirus-infected fish (Fig. 4). Notably, although the mRNA levels of transferrin and ferritin, both involved in iron metabolism with spleen and liver as the two main organs, increased in the liver after infection with both pathogens, they increased only in the spleen of *V. anguillarum*-infected animals (Figs. 5a and 5b).

**Figure 6:**
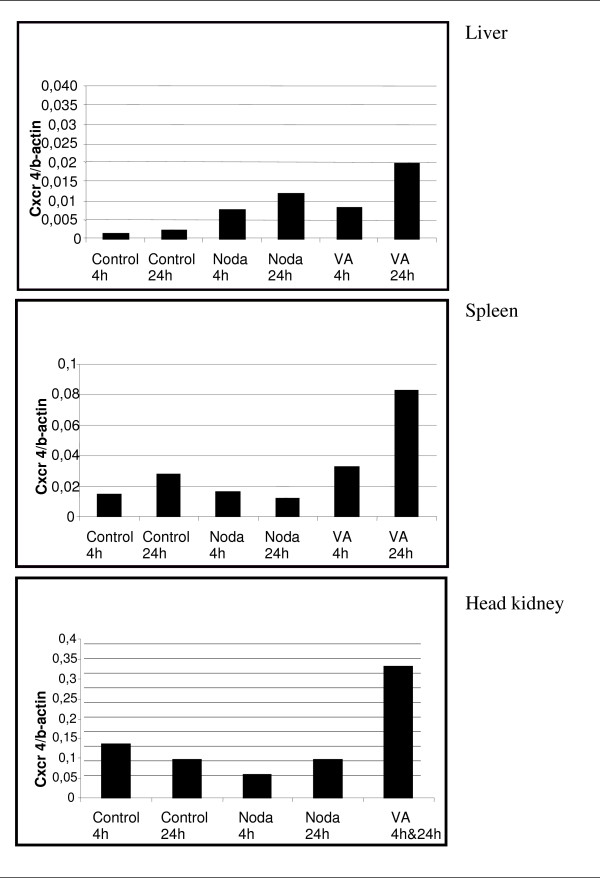
**Chemokine (c-x-c motif) receptor 4 (Cxcr 4) expression index of liver, spleen, and head kidney infected with Nodavirus or with *V. anguillarum *for 4 h and 24 h**. Infection for 4 h and 24 h of head kidney with *V. anguillarum *is pooled as not enough material was available. Each bar represents the mean of two technical duplicates of cDNA originating out of three individuals pooled prior to RNA extraction. Between the two technical replicates of Noda 4 h and of VA 24 h a greater variation was detected. The values of the two replicates of Noda 4 h are 0.014 and 0.00095 and the values of the two replicates of VA 24 h are 0.03 and 0.009.

**Figure 7:**
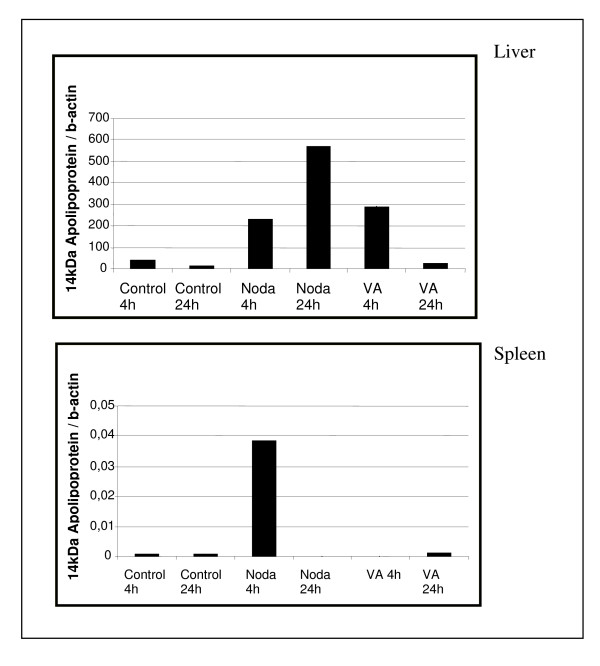
**14 KDa apolipoprotein expression index of liver infected with Nodavirus or with *V. anguillarum *for 4 h and 24 h**. Each bar represents the mean of two technical duplicates of cDNA originating out of three individuals pooled prior to RNA extraction.

**Figure 8:**
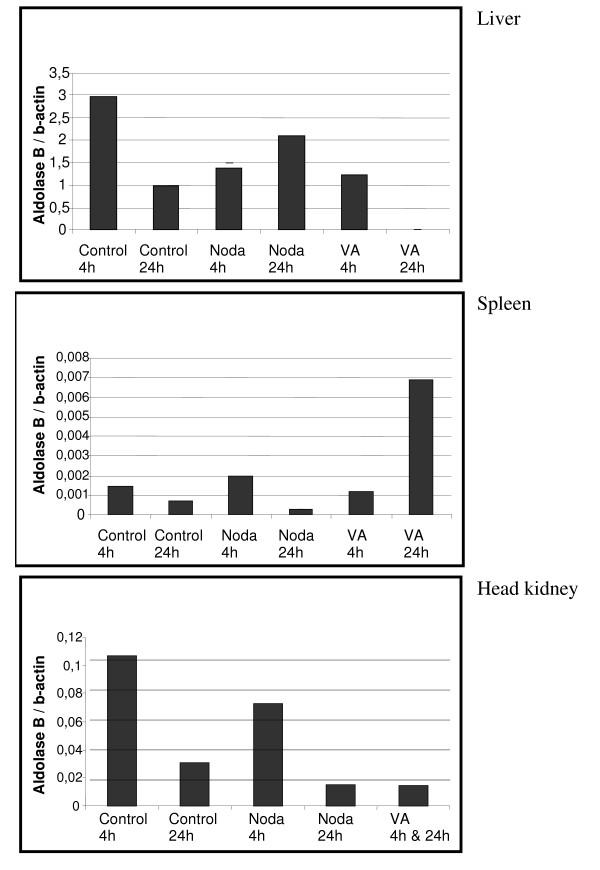
**Fructose-1,6-bisphosphatase aldolase B expression index of liver, spleen, and head kidney infected with Nodavirus or with *V. anguillarum *for 4 h and 24 h**. Infection for 4 h and 24 h of head kidney with *V. anguillarum *is pooled as not enough material was available. Each bar represents the mean of two technical of cDNA originating out of three individuals pooled prior to RNA extraction.

The mRNA levels of the chemokine receptor 4 were not affected or were slightly reduced in the head kidney and spleen of Nodavirus-infected fish but were considerably increased in these two tissues after *V. anguillarum *infection (Fig. 6). On the other hand, the mRNA levels of the 14 kDa apolipoprotein increased in the fish livers infected with both pathogens, but at 4 h and 24 h post-infection in the case of the Nodavirus and at 4 h post-infection in the case of *V. anguillarum *(Fig. 7). Here the expression in the liver is studied, as the liver is the major organ in the production of apoliprotein. Finally, although the mRNA levels of fructose-1,6-bisphosphate aldolase B decreased in the liver and head kidney following infection with both pathogens, they increased at 24 h post-infection in the spleen of *V. anguillarum*-infected fish (Fig. 8).

## Discussion

Although viral and bacterial infections are among the key challenges in fish aquaculture, nevertheless today the immune response of fish against *V. anguillarum *and Nodavirus remains largely unknown. Identification of genes involved in the immune response as well as the detection of differentially expressed genes between the two infections can make a significant contribution to future research leading to a better understanding of the biological system of immune response after fish infection. In the present study ten cDNA libraries, six from tissues infected with *V. anguillarum *and four from tissues infected with Nodavirus were analysed. Analysis of EST sequences coming from infected tissues will enhance the construction of an immune specific microarray chip containing already known transcripts involved in immune-related biological processes, such as the immune response as well as transcripts for which no annotation is available so far. Furthermore, transcripts indicating a higher expression level in one of the infections can be taken for future functional studies at RNA or DNA level as well as at protein level.

**Figure 9:**
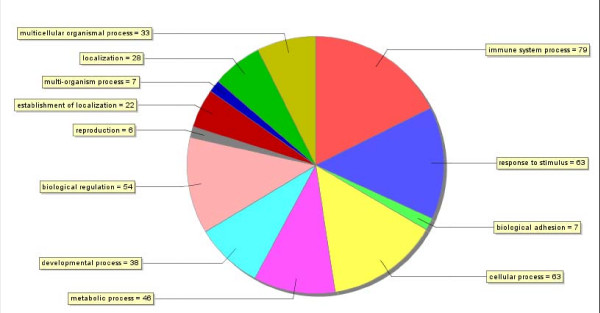
**GO categorization of only the EST sequences grouped into the category of immune system process**.

Over the past 30 years cDNA cloning for gene discovery and transcriptome analysis has become a very important molecular technique. Various techniques have been developed to address several scientific issues such as the cloning of rare transcripts, the construction of libraries with a wider cloning range, etc. (for review [26]). Construction of non-normalized libraries in the present study gave a first insight into the tissue-specific manner of transcript abundance according to their origin. Besides the possibility of identifying higher expressed transcripts, the percentages of unique sequences can also be assessed. In this study the redundancy of the cDNA libraries of liver, spleen and kidney infected with Nodavirus and *V. anguillarum *was in agreement with all three tissues (~33%, ~63% and ~38% respectively). This result is in line with other cDNA libraries of various fish species where the redundancy ranges between 40% and 60% depending on the tissues of origin [e.g. [12,13]]. Besides the identification and characterization of ESTs for components of the immune system, detection of microsatellite sequences will help in the completion of quantitative trait locus (QTL) scans currently being performed. Microsatellite sequences, also called Simple Sequence Repeats (SSR), are frequent in non-coding regions and are used as molecular markers. Detection of SSR within ESTs (exonic microsatellites or EST-SSRs) presents a shortcut to obtaining microsatellite markers. Since EST-SSRs are exonic they have two advantages over intergenic microsatellites. First, it is expected that their flanking regions are more conserved, so that the primers can be used even in related species, and second, it is assumed that they are in strong linkage disequilibrium with functionally important sites. Therefore they are frequently used in population genomics or in mapping of genes of economic significance identified as candidate markers for QTL and/or quantitative trait nucleotide (QTN). For EST similarity search in the present study a homologue of a known gene is defined as a cDNA whose similarity to a gene of any other organism in the database exceeds a certain fixed threshold. The identification of orthologues is outside the scope of this study. In total 1246 (41%) were assigned to a known transcript, with 79 (6%) categorized to the GO category "immune system process". Separate examination of these 79 transcripts by GO annotation reveals their involvement in 11 other categories of biological function (Fig. 9), with three dominant categories of response to stimulus, cellular process, and biological regulation. This collection should provide the base material for further research into understanding the immune response of European seabass as well as for the isolation of putative biomarkers.

### Similarity relationships

Comparison of predicted seabass genes compared to the genomes of zebrafish, medaka, tetraodon, stickleback and human (Fig. 2) showed that the majority of putative proteins were located in the centre. From separate examination of the different triads a bias towards the top and right sections is revealed. This bias is not unexpected as seabass is more closely related to medaka, zebrafish, stickleback and tetraodon. However it is worth noting, that the seabass cytochrome b seems to be more similar to human cytochrome b than to the tetraodon and medaka cytochrome b as shown in Fig. 2. This was not the case with stickleback and zebrafish cytochrome b. Interestingly, results from comparisons of putative proteins of the Atlantic halibut (*Hippoglossus hippoglossus*) with the human, zebrafish and tetraodon protein database showed that the halibut cytochrome c oxidase subunit 3 (Cox3) is more similar to human COX3 than the zebrafish and tetraodon Cox3 [13]. Comparison of predicted proteins with only the protein database of fish genomes shows a slight bias towards medaka and stickleback looking at the triad medaka, stickleback and tetraodon (Fig. 3A) and again a slight bias towards medaka and stickleback looking at the triad stickleback, medaka and zebrafish (Fig. 3B). These results give a first insight towards the evolution of immune related genes as the relatively equal distribution indicate that sequence variation between the clade Percomorpha is comparable to that between the clade Percomorpha and Ostariophysi.

### Expression analysis

For *in silico *expression analysis transcript appearing more than once in the cDNA libraries were selected and their relative abundance were submitted to expression analysis after Stekel et al. [22]. Validation of *in silico *analysis was performed by qPCR. Individual variation may be masked in this approach as pooling strategy was chosen for qPCR experiments. The differential expressed transcripts detected in the present study can be further put forward for analysis of individual expression pattern. Nonetheless in order to study individual expression pattern the sampling frame has to be extended. In the present study the pooling strategy for qPCR was chosen in order to show cross-method consistency. However since results are consistent between the two approaches, influence of between individuals variability in response to infection has been addressed to some extent. In addition total RNA for qPCR analysis was extracted out of different individuals than the once used for cDNA library construction and patterns appear to be consistent between the different samples for all the selected candidate genes, which reflect the robustness of the approach and the small, if any bias, contributed by individual outliers. *In silico *expression analysis revealed a number of genes for R > 6 that are considerably above the exponential curve (see Additional file 6). Genes with R > 6 can be considered as significant and thus are candidate genes for further studies. Several of those transcripts including transcripts involved in iron metabolism such as ferritin and transferrin are also reported as differential expressed genes in the catfish *Ictalurus punctatus *and *Ictalurus furcatus *infected with the gram negative bacterium *Edwardsiella ictaluri *[27,28]. One of the main mechanisms whereby gram-negative bacteria pathogens like *V. anguillarum *obtain iron is the use of free heme or heme proteins from the host tissues [29]. The heme uptake mechanisms are considered to contribute to *V. anguillarum *virulence in fish [29]. However, it is surprising that Nodavirus infection also resulted in the up-regulation of transferrin and ferritin expression, especially within 24 h of infection. The abundance of transferrin transcripts in Nodavirus-infected tissues may not be related to the alteration of the iron metabolism by the pathogen but rather to the ability of enzymatically cleaved forms of this protein to activate fish macrophages [30]. The specific alteration of iron metabolism by *V. anguillarum *infection is also supported by the higher abundance of transcripts coding for hepcidin, a major homeostatic regulator of iron metabolism [31], and for α and β chains of hemoglobin in *V. anguillarum*- than Nodavirus-infected livers (255 clones vs. 1 clone, respectively). The expression of Hepicidin after bacterial infection has been shown in seabass [32] as well as in several other fish species like the striped bass [33], the red sea bream [34], the catfish [35,36], the Atlantic halibut [37], the zebrafish [38], the rainbow trout [39] and the perch [40]. In this study the qPCR experiments confirmed the up-regulation of hepicidin in *D. labrax *after infection with *V. anguillarum *and showed in addition to this, that the expression of hepcidin might be considered as an excellent marker of bacterial infections, since it was up-regulated in all examined tissues of *V. anguillarum*-infected fish but unaffected in Nodavirus-infected tissues. Another interesting observation of the *in silico *gene expression analysis is the differential abundance of transcripts encoding the isoforms A and B glycolytic/gluconeogenic enzyme fructose-1,6-biphopshate aldolase in bacterial and viral infected tissues. Although the role played by this enzyme in the outcome of these infections is difficult to anticipate due to its dual role in glucose metabolism, these results suggest that the expression ratio between the two enzyme isoforms may be used as a good indicator of the type of infection in the European seabass. Thus, the up-regulation of the B isoform in the spleen exclusively by *V. anguillarum *might be considered another potential marker for this bacterial infection. Similarly, apolipoprotein A1 and 14 kDa apolipoprotein, two major components of high density lipoproteins (HDL) and synthesized in the fish liver [41], also show a differential expression in the liver of fish infected with *V. anguillarum *and Nodavirus following the time course and, therefore, they also may be good candidate indicators of the fish health status and/or the type of infection. The real-time PCR confirmed observations of *in silico *expression analysis and also revealed that the expression of the 14 kDa apolipoprotein and aldolase B in the spleen is an appropriate marker of Nodavirus and *V. anguillarum *infections, respectively. Previous studies in carp and medaka have also shown the involvement of apolipoproteins in the immune response [42,43]. Finally, the differential expression of one of the clear immune-related genes, the chemokine receptor 4, was also found to be a good putative marker for *V. anguillarum *infection. For assessment of variability of putative markers further studies looking at individuals, exposed to other environmental or pathogenic conditions are needed to exclude possible biological variability caused by infections.

## Conclusion

In this study we generated a collection of EST sequences from tissues of the European seabass infected with *V. anguillarum *and Nodavirus. We compared gene expression of different tissues after viral and pathogenic bacteria infection. A collection of 3075 unigenes was generated and candidate microsatellite sequences detected. Furthermore, comparisons of *D. labrax *transcripts with zebrafish, human, tetraodon, medaka and stickleback were performed. The majority of putative proteins were located in the centre with a bias towards the right sections, with *D. labrax *as expected being more closely related to the other fish species than to human. Comparison of putative *D. labrax *proteins was also performed among fish species. In this case a slight bias towards stickleback and medaka was observed when comparing medaka, stickleback and tetraodon and a slight bias towards stickleback and medaka was observed when comparing medaka, stickleback and zebrafish. Furthermore, *in silico *analysis of differential gene expression between the two infections based on EST sequences suggests a list of genes with a presumed function in the immune response of *D. labrax *revealing also the importance of looking at "non-classical" immune host proteins and emphasizing the significance of EST sequences generated from cDNA libraries of infected fish tissues. In addition, we show the power of sequencing cDNA sequences for expression analysis by performing real-time PCR experiments for transcripts with high, medium and low R-value. In view of new and high throughput sequence techniques detection of differential expression by measuring *in silico *the abundance of each transcript will enhance significantly the era of functional genomics. Furthermore *in silico *analysis in this study, followed by the confirmation with real-time PCR of potentially interested genes, has revealed some of them as potential biomarkers for bacterial and viral infections in fish.

## Methods

### Experimental condition and tissues collection

Two infections, one with Nodavirus strain 475-9/99 isolated from diseased sea bass [from the Instituto Zooprofilattico Sperimentale delle Venezie (Italy) [16]] and one with *V. anguillarum *strain R-82 (serogoup 01) [from the University of Santiago (Spain) [14]] were performed with seabass as previously described [14,16]. Tissues were taken 4 and 24 h post-infection. Three tissue types (spleen, liver and head kidney) of each experimental condition as well as peritoneal exudate, gill, intestine from *V. anguillarum *infection and brain from Nodavirus infection were selected and immediately frozen with liquid nitrogen. The experiments described comply with the guidelines of the European Union Council (86/609/EU) for the use of laboratory animals and have been approved by the Bioethical Committee of the University of Murcia (Spain) and the CSIC National Committee on Bioethics.

In brief; For Nodavirus infection fish were injected intramuscularly with 100 μl of nodavirus suspension in Minimum Essential Medium (MEM) (5.9 × 10^6 ^TCID_50 _ml^-1^) and placed at 25°C. Mock-infected control fish were injected with the medium alone, and maintained under the same experimental conditions. Three fish from each experimental and control groups were sampled 4 and 24 hours post-infection. Animals were sacrificed by anesthetic (MS-222) overdose and dissected. For the present study brain, spleen, head kidney and liver were sampled. For *V. anguillarum *infection fish were injected intraperitoneally (i.p.) with 1 ml of phosphate-buffered saline (PBS) alone or containing either 2 × 10^6 ^live or 10^8 ^formalin-killed *V. anguillarum *R82 cells (serogroup 01). Under these experimental conditions, about half of the fish were moribund at 24 h post-infection and all of them died within 48 h post-infection. Head kidney (bone marrow equivalent of fish) and peritoneal exudate cells were obtained 4 h and 24 h after bacterial challenge.

### RNA extraction

Total RNA was extracted using the NucleoSplin RNA II extraction kit (Machinery Nagel, Dueren, Germany). RNA quality was checked on EtBr stained agarose gels and RNA concentrations and purity were measured using a NanoDrop spectrophotometer. For library construction equal amounts of total RNA extracted out of infected tissues (4 h and 24 h) were pooled. For qPCR experiments total RNA was freshly extracted out of infected tissues originating from three different individuals pooled prior to RNA extraction (liver, spleen and head kidney) with 4 h and 24 h post-infection.

### cDNA library construction

All libraries were constructed from total RNA using the Creator SMART cDNA library construction kit (BD Bioscience-Clontech, Mountain View, Canada) using the LD PCR based method. Between 20 and 22 PCR cycles were performed before size separation of inserts. cDNA fragments > 600 bp were selected and directionally ligated at the restriction site Sfi1 of the pDNR-lib vector (BD Clontech) or the pal 32 vector. Plasmids were transformed into *E. coli *strain DH10B (Invitrogen) by electroporation. The libraries were tested for the presence and the size of insert by PCR using two primer pairs. For the libraries constructed with pal 32 vector, the primer pair pal 32 FOR: 5'-CTCGGGAAGCGCGCCATT-3' and pal 32, REV: 5'-TAATACGACTCACTATAGGGC-3' were used. For the libraries constructed with pDNR-lib vector pDNR FOR: 5'-TAAAACGACGGCCAGTA-3' pDNR REV: 5'-GAAACAGCTATGACCATGTTC-3' were used. The products were run on an EtBr stained agarose gel.

### DNA sequencing

After plasmid preparation, dideoxy-temination DNA cycle sequencing was performed using the BigDye 3.1 sequencing method and the pDNR FOR (5'-TAAAACGACGGCCAGTA-3') primer. The sequences were run on an ABI 3730 XL sequencer at MPI Molecular Genetics, Berlin.

### Sequence analysis

The raw sequence reads were quality-trimmed and vector- and poly-A-clipped using PREGAP4 [18]. Clustering (grouping of clones related to one another by sequence homology) was performed using the software SeqManII (DNAstar Inc.). After clustering the term 'contig' is used to describe the sequence obtained from one cluster (the sequences of a cluster can be collapsed into a single, non-redundant sequence) and the term 'singleton' describes sequences appearing only once in the entire dataset. The set of sequences obtained by merging contigs and singletons are named as unique sequences.

### Simple Sequence Repeats (SSR) in EST sequences

*In silico *mining for repeat motifs within the obtained unique sequences was perfomed with the programme Msatfinder [19].

### Homology search and GO annotation

Gene Ontology (GO) category (Biological process) was assigned after BLASTX search of 3075 unique EST sequences using BLAST2GO. Threshold cutoff was at E-value 1e^-3 ^and the alignment length of 33 amino acids (aa).

### Similarity relationships

The unique sequences from all seabass libraries were submitted to BLASTX similarity searches [20] against the zebrafish, tetraodon, stickleback, medaka and human predicted proteomes (downloadable from ). For each database the highest BLAST scores (bit score values) in excess of 50 were retained. Relative similarities between triads were visualized as a triangular plot generated by the SimiTri software [21].

### Expression analysis

#### In silico

All sequences of each cDNA library were submitted to BLASTX and BLASTN searches [20]. Transcripts appearing more than once in the cDNA libraries were selected for *in silico *expression analysis after Stekel *et al*. [22]. In brief, this method allows the comparison of gene expression in any number of libraries in order to identify differential expressed genes. The method uses a single statistical test to describe the extent to which a gene is differentially expressed between libraries by a log likelihood ratio statistic and tends asymptotically to a χ^2 ^distribution [22]. For real-time PCR experiments transcripts with high, medium and low R-value were selected.

#### Real-time PCR

Gene expression was assessed by real-time PCR (qPCR) in spleen, head kidney and liver at 4 h and 24 h post-infection. RNAs out of three animals pooled prior to RNA extraction were isolated as described above and were used to obtain cDNA by the Superscript II Reverse Transcriptase and oligo (dT)12–18 primer (Invitrogen) following the manufacturer's instructions. Quantitative PCR assays were performed using the 7300 real-time PCR System (Applied Biosystems) with specific primers (Table 3). Each primer (0.5 μl; 10 μM) and the cDNA template (1 μl) were mixed with 12.5 μl of SYBR green PCR master mix (Applied Biosystems) in a final volume of 25 μl. The standard cycling conditions were 95°C for 10 min. followed by 40 cycles of 95°C 15 s and 60°C 1 min. For all reactions two technical duplicates were performed. The comparative CT method (2-ΔΔ CT method) was used to determine the expression level of analysed genes [23]. After evaluation of β-actin as a suitable reference gene for this study in seabass (data not shown) the expression of the candidate genes was normalized. The use of β-actin as a suitable reference gene was also shown in other fish studies [e.g. [24,25]].

**Table 3:** Real-time primer sequences

**Name**	**F/R**	**Sequence 5'-3'**
β-actin	Forward	GTGCGTGACATCAAGGAGAA
β-actin	Reverse	GCTGGAAGGTGGACAGAGAG
Apoliprot	Forward	ATACGTCCTGGCACTGATCC
Apoliprot	Reverse	AGCCTGACCTTGCTCACTGT
Chemokin-R4	Forward	TCAAAACGATGACGGACAAG
Chemokin-R4	Reverse	ACACGCTGCTGTACAGGTTG
Transferrin	Forward	CTGGGAAGTGTGGTCTGGTT
Transferrin	Reverse	CAAGACCTCTTGCCCTTCAG
Ferritin_HC	Forward	ATGCACAAGCTCTGCTCTGA
Ferritin_HC	Reverse	TTTGCCCAGGGTGTGTTTAT
Hepcidin-Prec	Forward	CCAGTCACTGAGGTGCAAGA
Hepcidin-Prec	Reverse	TCAGAACCTGCAGCAGACAC
Aldolase-B	Forward	TGACATTGCTCAGAGGATCG
Aldolase-B	Reverse	AGTTGGACATGGAGGGACTG

## Authors' contributions

ES contributed to EST production, performed analysis and the conception, design and manuscript writing. PS performed infections with VA and tissue sampling and contributed to RNA extractions. LP performed qPCR experiments. VM conceived the study, contributed to the conception and design of the project and also to the manuscript writing. JM contributed to the conception and design of the project. AF contributed to conception and design of the project and helped to draft the manuscript. BN conceived qPCR experiments, Nodavirus infections and tissue sampling and contributed to conception and design of the project and helped to draft the manuscript. VT and RR created the cDNA libraries and performed the sequencing of the clones. AM contributed to conception and design of the project and helped to draft the manuscript. GK conceived the study, contributed to the conception and design of the project and also to the manuscript writing.

## Supplementary Material

Additional file 1 **Appendix 1**. Catalogue of all unique sequences obtained from all constructed cDNA libraries in this study. Click here for file

Additional file 2 **Appendix 2**. EST-SSRs identified among EST sequences of all cDNA libraries constructed in this study. Click here for file

Additional file 3 **Appendix 3**. EST sequences (after clustering of all 3075) grouped into GO group Immune system process. Click here for file

Additional file 4 **Appendix 4**. A: CDNA sequences of hepicidin precursor showing putative isoforms B: CDNA showing microsatellite sequence with in silico SSR polymorphism after alignment of 8 sequences and C: sequences of cysteine-rich protein 1-I showing putative alternative splicing polyadenylations. Click here for file

Additional file 5 **Appendix 5**. Calculated R-values for contigs resulting from the EST sequencing of the cDNA library of spleen, liver and kidney infected with Nodavirus and Vibrio anguillarum. Click here for file

Additional file 6 **Appendix 6**. The number of genes for a given value or the test statistic R is plotted as a function of R. The data falling within 1 < R < 6 are decrees exponential curve, and decreasing exponentially with R. The slope is -1.081 with significance at the 5% level of 0.013 and is therefore not significantly different from -1 at 5% significance. When R > 6, the number of genes is above this exponential curve. Click here for file
