# Supplementary material for: Profiling of infection specific mRNA transcripts of the European seabass Dicentrarchus labrax
Source: BMC Genomics. 2009 Apr 10;10:157. doi: 10.1186/1471-2164-10-157 (PMC2674461; doi:10.1186/1471-2164-10-157)
Supplement: Additional file 2 — Appendix 2. EST-SSRs identified among EST sequences of all cDNA libraries constructed in this study. [file 1471-2164-10-157-S2.doc]

**Appendix 2**: repeat motifs found in unique EST sequences

| **sequence_identifier** | **repeat** | **Sequences identifier** | **repeat** |
| --- | --- | --- | --- |
| Contig_1 | (ac)14 | Contig_1330 | (ctga)5 |
| Contig_1 | (ac)5 | Contig_136 | (tg)12 |
| Contig_1 | (ca)12 | Contig_1367 | (ac)5 |
| Contig_1 | (ca)16 | Contig_137 | (ac)11 |
| Contig_1 | (ca)38 | Contig_138 | (at)5 |
| Contig_1 | (ca)42 | Contig_138 | (ca)11 |
| Contig_1 | (ca)5 | Contig_138 | (gtt)8 |
| Contig_1 | (ca)8 | Contig_138 | (ta)6 |
| Contig_1 | (ca)8 | Contig_138 | (tg)6 |
| Contig_10 | (gt)13 | Contig_1388 | (ta)7 |
| Contig_10 | (gt)14 | Contig_1397 | (ga)6 |
| Contig_10 | (gt)18 | Contig_141 | (ac)8 |
| Contig_10 | (gt)6 | Contig_141 | (at)9 |
| Contig_10 | (gt)7 | Contig_141 | (ca)9 |
| Contig_10 | (tg)11 | Contig_1417 | (ct)6 |
| Contig_10 | (tg)11 | Contig_1417 | (ga)6 |
| Contig_10 | (tg)17 | Contig_142 | (gt)12 |
| Contig_10 | (tg)5 | Contig_142 | (tg)5 |
| Contig_10 | (tg)5 | Contig_1420 | (ac)5 |
| Contig_10 | (tg)7 | Contig_143 | (ac)8 |
| Contig_100 | (ac)14 | Contig_143 | (gt)7 |
| Contig_100 | (agc)5 | Contig_143 | (tg)5 |
| Contig_100 | (agg)5 | Contig_143 | (tg)9 |
| Contig_100 | (ct)9 | Contig_1437 | (agga)11 |
| Contig_1005 | (agg)5 | Contig_145 | (ac)7 |
| Contig_1015 | (gt)6 | Contig_1459 | (ac)5 |
| Contig_102 | (tg)16 | Contig_146 | (ca)10 |
| Contig_103 | (ac)16 | Contig_146 | (ca)5 |
| Contig_1045 | (ata)5 | Contig_150 | (tg)11 |
| Contig_105 | (ac)15 | Contig_1500 | (ca)5 |
| Contig_1055 | (tc)6 | Contig_1514 | (ga)5 |
| Contig_1071 | (ctc)8 | Contig_1517 | (tgc)5 |
| Contig_1079 | (ag)5 | Contig_1557 | (gt)5 |
| Contig_1079 | (ag)5 | Contig_1557 | (gt)5 |
| Contig_1079 | (ag)5 | Contig_1557 | (gt)5 |
| Contig_1079 | (ga)7 | Contig_1558 | (ca)5 |
| Contig_1082 | (ag)7 | Contig_1558 | (tg)5 |
| Contig_1096 | (ttta)5 | Contig_1559 | (gt)5 |
| Contig_1097 | (aaat)5 | Contig_156 | (gt)8 |
| Contig_11 | (gt)6 | Contig_156 | (gt)9 |
| Contig_11 | (tg)10 | Contig_156 | (tg)6 |
| Contig_11 | (tg)26 | Contig_159 | (ac)10 |
| Contig_11 | (tg)8 | Contig_162 | (cag)6 |
| Contig_1105 | (ct)5 | Contig_163 | (tg)8 |
| Contig_1109 | (tta)5 | Contig_164 | (tg)11 |
| Contig_1118 | (tcc)7 | Contig_164 | (tg)5 |
| Contig_1119 | (tcc)6 | Contig_165 | (ac)8 |
| Contig_1120 | (gag)6 | Contig_166 | (ctg)5 |
| Contig_1121 | (cct)5 | Contig_167 | (gcgt)5 |
| Contig_1139 | (gt)5 | Contig_167 | (gt)9 |
| Contig_114 | (gt)14 | Contig_1670 | (tg)6 |
| Contig_114 | (gt)7 | Contig_1671 | (tg)6 |
| Contig_1141 | (gat)5 | Contig_169 | (tg)10 |
| Contig_1152 | (at)5 | Contig_170 | (gt)10 |
| Contig_116 | (atct)10 | Contig_1715 | (gt)5 |
| Contig_116 | (gt)5 | Contig_172 | (gt)10 |
| Contig_116 | (gt)7 | Contig_1724 | (tg)5 |
| Contig_116 | (gt)8 | Contig_173 | (ca)10 |
| Contig_116 | (tg)7 | Contig_1784 | (ca)5 |
| Contig_119 | (ac)13 | Contig_1785 | (tg)5 |
| Contig_1195 | (ca)5 | Contig_18 | (ca)31 |
| Contig_12 | (ta)5 | Contig_180 | (ac)7 |
| Contig_120 | (ac)13 | Contig_180 | (gt)6 |
| Contig_120 | (tg)5 | Contig_180 | (tg)5 |
| Contig_121 | (ca)11 | Contig_182 | (ca)9 |
| Contig_121 | (tc)12 | Contig_184 | (gt)5 |
| Contig_123 | (gt)6 | Contig_184 | (tg)8 |
| Contig_123 | (gt)7 | Contig_185 | (ac)7 |
| Contig_123 | (gt)7 | Contig_1876 | (tg)5 |
| Contig_123 | (tg)9 | Contig_19 | (ac)36 |
| Contig_1247 | (at)5 | Contig_1904 | (ac)5 |
| Contig_125 | (ca)13 | Contig_191 | (tca)8 |
| Contig_1272 | (agaaa)5 | Contig_1917 | (ca)5 |
| Contig_1279 | (ca)5 | Contig_1927 | (ac)5 |
| Contig_1279 | (ta)5 | Contig_194 | (ca)8 |
| Contig_129 | (ca)13 | Contig_194 | (taa)7 |
| Contig_129 | (gtct)14 | Contig_196 | (gt)8 |
| Contig_13 | (ac)10 | Contig_196 | (tg)5 |
| Contig_13 | (ac)16 | Contig_199 | (ca)9 |
| Contig_13 | (ac)6 | Contig_2 | (ac)10 |
| Contig_13 | (ac)7 | Contig_2 | (ac)5 |
| Contig_13 | (ca)15 | Contig_2 | (ac)7 |
| Contig_13 | (ca)5 | Contig_2 | (ac)9 |
| Contig_13 | (ca)5 | Contig_2 | (ca)13 |
| Contig_130 | (tg)5 | Contig_2 | (ca)16 |
| Contig_130 | (tg)6 | Contig_2 | (ca)5 |
| Contig_130 | (tg)7 | Contig_2 | (ca)6 |
| Contig_130 | (tg)8 | Contig_2 | (ca)7 |
| Contig_130 | (tg)8 | Contig_2 | (ca)8 |
| Contig_132 | (gt)11 | Contig_2 | (ca)8 |
| Contig_133 | (gt)13 | Contig_2 | (ca)9 |
|  |  | Contig_2 | (ca)9 |
| Contig_2 | (tc)5 | Contig_2457 | (ag)5 |
| Contig_2007 | (ca)5 | Contig_2468 | (tc)5 |
| Contig_2012 | (gt)5 | Contig_2491 | (ga)5 |
| Contig_203 | (tg)9 | Contig_25 | (tg)36 |
| Contig_2070 | (ca)5 | Contig_250 | (ag)5 |
| Contig_2076 | (ca)5 | Contig_250 | (ag)6 |
| Contig_21 | (tat)7 | Contig_250 | (ga)5 |
| Contig_210 | (ac)6 | Contig_2506 | (ac)6 |
| Contig_210 | (ac)7 | Contig_2539 | (at)5 |
| Contig_210 | (ac)8 | Contig_2539 | (ga)5 |
| Contig_211 | (ac)5 | Contig_254 | (ac)5 |
| Contig_212 | (tg)8 | Contig_254 | (ac)7 |
| Contig_213 | (ct)7 | Contig_254 | (ta)6 |
| Contig_213 | (gt)8 | Contig_2553 | (agt)5 |
| Contig_214 | (ac)5 | Contig_2565 | (ct)5 |
| Contig_214 | (ca)7 | Contig_257 | (ac)7 |
| Contig_214 | (tctt)5 | Contig_2577 | (ga)5 |
| Contig_215 | (gt)8 | Contig_259 | (ca)7 |
| Contig_216 | (ca)8 | Contig_26 | (aat)15 |
| Contig_218 | (tg)5 | Contig_26 | (ac)13 |
| Contig_218 | (tg)5 | Contig_26 | (ag)9 |
| Contig_218 | (tg)6 | Contig_26 | (at)6 |
| Contig_2185 | (ct)5 | Contig_26 | (ga)5 |
| Contig_220 | (at)5 | Contig_26 | (ga)6 |
| Contig_222 | (tg)8 | Contig_26 | (gt)6 |
| Contig_223 | (ac)5 | Contig_26 | (gt)6 |
| Contig_223 | (ac)5 | Contig_26 | (gt)8 |
| Contig_224 | (ac)8 | Contig_26 | (ta)5 |
| Contig_224 | (ca)6 | Contig_26 | (tg)12 |
| Contig_226 | (tg)5 | Contig_260 | (tg)6 |
| Contig_227 | (ta)6 | Contig_261 | (ac)7 |
| Contig_227 | (tg)7 | Contig_262 | (gt)7 |
| Contig_2274 | (ga)5 | Contig_2621 | (ag)5 |
| Contig_228 | (ca)5 | Contig_2621 | (ct)5 |
| Contig_228 | (tca)6 | Contig_263 | (gt)5 |
| Contig_2289 | (ag)5 | Contig_263 | (tg)5 |
| Contig_23 | (ac)34 | Contig_2630 | (tg)5 |
| Contig_23 | (tg)5 | Contig_2634 | (ta)5 |
| Contig_230 | (gt)5 | Contig_264 | (gt)6 |
| Contig_231 | (ca)6 | Contig_2657 | (ac)5 |
| Contig_232 | (at)8 | Contig_2663 | (ac)6 |
| Contig_233 | (tg)7 | Contig_2679 | (ta)6 |
| Contig_2368 | (gt)5 | Contig_275 | (taa)12 |
| Contig_24 | (tg)27 | Contig_2817 | (ga)5 |
| Contig_24 | (tg)5 | Contig_2858 | (ag)6 |
| Contig_2409 | (ggt)5 | Contig_2858 | (cca)5 |
|  |  |  |  |
| Contig_2881 | (tc)5 | Contig_392 | (at)7 |
| Contig_289 | (ta)5 | Contig_392 | (ta)5 |
| Contig_2910 | (ga)5 | Contig_395 | (gt)7 |
| Contig_2919 | (gt)5 | Contig_396 | (tgtt)7 |
| Contig_2924 | (ct)5 | Contig_398 | (gt)5 |
| Contig_2942 | (tg)5 | Contig_399 | (aca)12 |
| Contig_2957 | (tg)5 | Contig_4 | (ac)30 |
| Contig_2965 | (ag)5 | Contig_4 | (ac)5 |
| Contig_2965 | (ga)5 | Contig_4 | (ca)10 |
| Contig_3027 | (gt)5 | Contig_4 | (ca)10 |
| Contig_304 | (ta)9 | Contig_4 | (ca)14 |
| Contig_3046 | (at)6 | Contig_4 | (ca)19 |
| Contig_307 | (ga)5 | Contig_4 | (ca)25 |
| Contig_31 | (gt)25 | Contig_4 | (ca)8 |
| Contig_31 | (tg)6 | Contig_4 | (ca)9 |
| Contig_32 | (ac)10 | Contig_401 | (ttg)8 |
| Contig_32 | (ac)15 | Contig_402 | (aca)5 |
| Contig_330 | (aaacta)5 | Contig_402 | (caa)8 |
| Contig_331 | (ca)5 | Contig_402 | (cag)5 |
| Contig_34 | (tg)13 | Contig_402 | (cag)5 |
| Contig_34 | (tg)18 | Contig_402 | (gca)10 |
| Contig_348 | (ttc)12 | Contig_405 | (caa)10 |
| Contig_35 | (ac)26 | Contig_405 | (ct)10 |
| Contig_351 | (ca)8 | Contig_406 | (aat)7 |
| Contig_351 | (ta)5 | Contig_41 | (tg)24 |
| Contig_357 | (gaa)8 | Contig_412 | (gag)5 |
| Contig_36 | (gt)25 | Contig_412 | (ttg)13 |
| Contig_368 | (tg)5 | Contig_414 | (gt)6 |
| Contig_37 | (ac)17 | Contig_414 | (tgt)11 |
| Contig_37 | (ca)12 | Contig_421 | (caa)8 |
| Contig_371 | (tg)8 | Contig_422 | (ttg)8 |
| Contig_374 | (gt)7 | Contig_425 | (gt)6 |
| Contig_376 | (ac)5 | Contig_426 | (tgt)6 |
| Contig_376 | (ac)6 | Contig_428 | (gt)5 |
| Contig_378 | (ga)7 | Contig_439 | (ct)7 |
| Contig_378 | (gt)6 | Contig_44 | (ga)5 |
| Contig_378 | (tg)7 | Contig_44 | (gt)14 |
| Contig_379 | (gtt)6 | Contig_44 | (gt)5 |
| Contig_382 | (tc)6 | Contig_44 | (gt)6 |
| Contig_386 | (tg)8 | Contig_44 | (gt)6 |
| Contig_387 | (gt)8 | Contig_44 | (tg)8 |
| Contig_388 | (ca)7 | Contig_441 | (ac)5 |
| Contig_39 | (gt)20 | Contig_443 | (at)8 |
| Contig_39 | (gtgc)6 | Contig_448 | (tg)6 |
| Contig_390 | (ac)6 | Contig_458 | (tg)5 |
| Contig_391 | (tg)5 | Contig_46 | (tg)22 |
|  |  |  |  |
| Contig_460 | (ca)6 | Contig_522 | (ac)6 |
| Contig_461 | (gt)5 | Contig_523 | (ac)6 |
| Contig_461 | (gt)5 | Contig_523 | (ca)5 |
| Contig_461 | (gt)5 | Contig_523 | (ct)5 |
| Contig_461 | (tg)5 | Contig_525 | (ac)5 |
| Contig_461 | (tg)6 | Contig_526 | (tg)6 |
| Contig_462 | (cag)8 | Contig_53 | (at)7 |
| Contig_462 | (gt)6 | Contig_53 | (ca)21 |
| Contig_464 | (ct)5 | Contig_530 | (ga)5 |
| Contig_464 | (gt)5 | Contig_532 | (ga)5 |
| Contig_464 | (tg)5 | Contig_537 | (agc)7 |
| Contig_465 | (ag)6 | Contig_549 | (cct)6 |
| Contig_465 | (gt)5 | Contig_550 | (aag)7 |
| Contig_466 | (gt)5 | Contig_56 | (gt)20 |
| Contig_466 | (gt)6 | Contig_561 | (at)6 |
| Contig_468 | (tg)5 | Contig_567 | (tat)10 |
| Contig_47 | (ct)11 | Contig_568 | (aat)11 |
| Contig_47 | (ct)9 | Contig_569 | (aat)10 |
| Contig_47 | (gt)18 | Contig_57 | (ct)5 |
| Contig_471 | (gt)7 | Contig_570 | (att)10 |
| Contig_48 | (tg)22 | Contig_572 | (aat)8 |
| Contig_482 | (tg)5 | Contig_573 | (att)7 |
| Contig_484 | (ac)5 | Contig_574 | (att)7 |
| Contig_487 | (ga)5 | Contig_575 | (tta)6 |
| Contig_5 | (ac)37 | Contig_576 | (aat)6 |
| Contig_5 | (ac)5 | Contig_577 | (att)6 |
| Contig_5 | (ac)6 | Contig_578 | (att)6 |
| Contig_5 | (ac)6 | Contig_579 | (tat)6 |
| Contig_5 | (ac)8 | Contig_58 | (ac)18 |
| Contig_5 | (ca)5 | Contig_580 | (ata)5 |
| Contig_5 | (ca)7 | Contig_591 | (ac)6 |
| Contig_5 | (ca)9 | Contig_594 | (ag)5 |
| Contig_5 | (tgt)11 | Contig_594 | (ga)12 |
| Contig_50 | (gt)21 | Contig_596 | (aaag)6 |
| Contig_509 | (tg)6 | Contig_596 | (ag)19 |
| Contig_51 | (gt)17 | Contig_596 | (ag)5 |
| Contig_51 | (tg)10 | Contig_596 | (ag)8 |
| Contig_510 | (ag)13 | Contig_597 | (ct)21 |
| Contig_510 | (ag)5 | Contig_598 | (ct)26 |
| Contig_510 | (agcg)5 | Contig_599 | (ga)16 |
| Contig_510 | (ga)13 | Contig_6 | (ca)16 |
| Contig_510 | (ga)5 | Contig_6 | (ca)5 |
| Contig_510 | (gt)5 | Contig_600 | (acag)5 |
| Contig_516 | (cgga)5 | Contig_600 | (acag)5 |
| Contig_516 | (ctt)6 | Contig_600 | (ag)5 |
| Contig_516 | (tg)5 | Contig_600 | (ag)5 |
|  |  |  |  |
| Contig_600 | (ag)5 | Contig_7 | (ca)18 |
| Contig_600 | (ag)5 | Contig_7 | (ca)5 |
| Contig_600 | (ag)7 | Contig_7 | (ca)56 |
| Contig_600 | (ag)8 | Contig_702 | (tc)7 |
| Contig_600 | (ag)8 | Contig_71 | (ac)16 |
| Contig_600 | (ga)6 | Contig_71 | (ga)5 |
| Contig_600 | (ga)8 | Contig_72 | (ca)18 |
| Contig_601 | (ag)9 | Contig_721 | (ac)5 |
| Contig_602 | (ag)9 | Contig_722 | (tc)7 |
| Contig_603 | (tc)9 | Contig_724 | (ag)7 |
| Contig_605 | (aat)5 | Contig_725 | (tca)7 |
| Contig_607 | (aat)5 | Contig_725 | (ttc)6 |
| Contig_608 | (att)5 | Contig_726 | (gat)9 |
| Contig_612 | (gca)7 | Contig_727 | (atg)9 |
| Contig_613 | (at)6 | Contig_728 | (gat)9 |
| Contig_613 | (ta)9 | Contig_729 | (atc)9 |
| Contig_614 | (at)9 | Contig_73 | (tga)10 |
| Contig_615 | (gt)5 | Contig_730 | (cat)8 |
| Contig_616 | (ta)11 | Contig_731 | (cat)5 |
| Contig_619 | (ta)5 | Contig_732 | (gaa)8 |
| Contig_62 | (ac)17 | Contig_732 | (gt)5 |
| Contig_62 | (tg)5 | Contig_733 | (aag)7 |
| Contig_620 | (at)6 | Contig_734 | (gttt)5 |
| Contig_626 | (ct)5 | Contig_74 | (tg)16 |
| Contig_634 | (ct)5 | Contig_74 | (ttc)5 |
| Contig_639 | (tc)7 | Contig_743 | (gag)10 |
| Contig_640 | (tg)6 | Contig_744 | (tcc)8 |
| Contig_641 | (gt)5 | Contig_745 | (ca)5 |
| Contig_641 | (ttga)5 | Contig_746 | (gga)5 |
| Contig_642 | (gac)6 | Contig_747 | (tcc)5 |
| Contig_647 | (ga)5 | Contig_760 | (gt)5 |
| Contig_648 | (ta)5 | Contig_767 | (tg)6 |
| Contig_65 | (ag)7 | Contig_775 | (cct)5 |
| Contig_65 | (gt)8 | Contig_78 | (ta)5 |
| Contig_65 | (tg)15 | Contig_781 | (ta)5 |
| Contig_655 | (ta)5 | Contig_782 | (ac)6 |
| Contig_674 | (ga)7 | Contig_782 | (gt)5 |
| Contig_675 | (at)6 | Contig_794 | (agat)5 |
| Contig_69 | (ac)11 | Contig_794 | (gata)7 |
| Contig_69 | (ca)12 | Contig_8 | (tg)25 |
| Contig_69 | (ga)5 | Contig_8 | (tg)45 |
| Contig_692 | (aga)5 | Contig_812 | (ca)5 |
| Contig_7 | (ac)6 | Contig_812 | (ta)7 |
| Contig_7 | (ac)9 | Contig_819 | (ga)6 |
| Contig_7 | (ac)9 | Contig_820 | (tg)5 |
| Contig_7 | (ca)13 | Contig_839 | (at)11 |
| Contig_989 | (tc)7 | Contig_988 | (ggt)6 |
| Contig_840 | (tg)5 | Contig_954 | (gaa)5 |
| Contig_845 | (tg)6 | Contig_955 | (aag)5 |
| Contig_85 | (tctgtg)5 | Contig_960 | (ga)5 |
| Contig_85 | (tg)13 | Contig_97 | (tcc)5 |
| Contig_85 | (tg)5 |  |  |
| Contig_852 | (cag)8 |  |  |
| Contig_853 | (tgc)7 |  |  |
| Contig_854 | (gca)7 |  |  |
| Contig_855 | (gct)5 |  |  |
| Contig_856 | (cag)5 |  |  |
| Contig_857 | (tgc)5 |  |  |
| Contig_858 | (at)6 |  |  |
| Contig_86 | (ca)15 |  |  |
| Contig_860 | (ta)5 |  |  |
| Contig_871 | (at)5 |  |  |
| Contig_88 | (ca)15 |  |  |
| Contig_88 | (ct)5 |  |  |
| Contig_88 | (tc)6 |  |  |
| Contig_892 | (ag)5 |  |  |
| Contig_9 | (ac)10 |  |  |
| Contig_9 | (ac)13 |  |  |
| Contig_9 | (ac)15 |  |  |
| Contig_9 | (ac)5 |  |  |
| Contig_9 | (ac)67 |  |  |
| Contig_9 | (ca)12 |  |  |
| Contig_900 | (gaa)5 |  |  |
| Contig_91 | (ag)5 |  |  |
| Contig_91 | (at)7 |  |  |
| Contig_91 | (gt)15 |  |  |
| Contig_912 | (tg)5 |  |  |
| Contig_915 | (tta)5 |  |  |
| Contig_916 | (ct)5 |  |  |
| Contig_92 | (tg)16 |  |  |
| Contig_92 | (tg)7 |  |  |
| Contig_922 | (at)5 |  |  |
| Contig_93 | (ac)14 |  |  |
| Contig_93 | (ca)7 |  |  |
| Contig_94 | (ta)5 |  |  |
| Contig_948 | (aga)5 |  |  |
| Contig_952 | (aag)5 |  |  |
| Contig_953 | (aga)5 |  |  |
|  |  |  |  |
