# Supplementary figures and images for: Profiling of infection specific mRNA transcripts of the European seabass Dicentrarchus labrax
Source: BMC Genomics. 2009 Apr 10;10:157. doi: 10.1186/1471-2164-10-157 (PMC2674461; doi:10.1186/1471-2164-10-157)

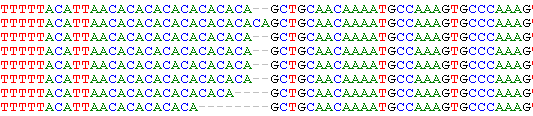


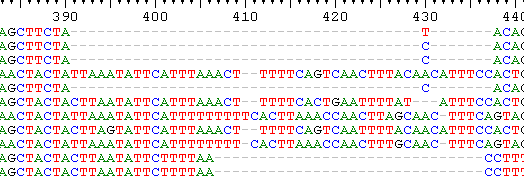


**C:**

**B:**

**A:**

Supplement: Additional file 4 — Appendix 4. A: CDNA sequences of hepicidin precursor showing putative isoforms B: CDNA showing microsatellite sequence with in silico SSR polymorphism after alignment of 8 sequences and C: sequences of cysteine-rich protein 1-I showing putative alternative splicing polyadenylations. [file 1471-2164-10-157-S4.doc]
