# Supplementary material for: Profiling of infection specific mRNA transcripts of the European seabass Dicentrarchus labrax
Source: BMC Genomics. 2009 Apr 10;10:157. doi: 10.1186/1471-2164-10-157 (PMC2674461; doi:10.1186/1471-2164-10-157)
Supplement: Additional file 6 — Appendix 6. The number of genes for a given value or the test statistic R is plotted as a function of R. The data falling within 1 < R < 6 are decrees exponential curve, and decreasing exponentially with R. The slope is -1.081 with significance at the 5% level of 0.013 and is therefore not significantly different from -1 at 5% significance. When R > 6, the number of genes is above this exponential curve. [file 1471-2164-10-157-S6.doc]

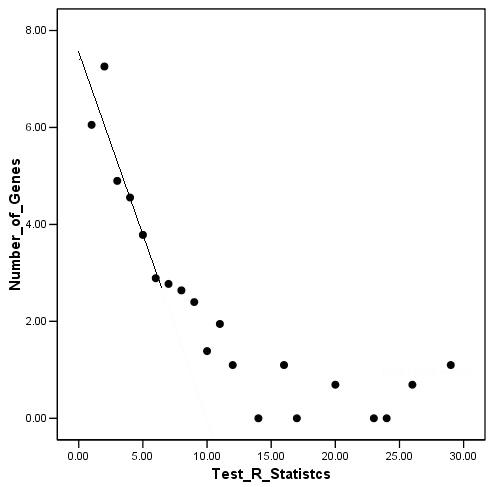


| Model |  | Unstandardized Coefficients | | Standardized Coefficients | t | Sig. |
| --- | --- | --- | --- | --- | --- | --- |
|  |  | B | Std. Error | Beta |  |  |
| 1 | (Constant) | 8.805 | 1.289 |  | 6.830 | .002 |
|  | VAR00003 | -1.081 | .252 | -.906 | -4.286 | .013 |

a Dependent Variable: VAR00001

**B=-1.081 significance at the 5% level :0.013**
